# Climate change: north and south EU economies—an application of dynamic asymmetric panel data models

**DOI:** 10.1007/s11356-022-22907-y

**Published:** 2022-09-16

**Authors:** Christos Adam, Periklis Drakos

**Affiliations:** Department of Economics, School of Social Sciences, University of Crete, 74100 Rethymno, Crete Greece

**Keywords:** Asymmetries, Climate change, European Union, Economic growth, Dynamic panel data, Pollution

## Abstract

The need for a cleaner environment and wealthier economies has been highly recognized by European Union (EU) policymakers of the last years, as evidenced by the creation of a plethora of laws and regulations for reducing carbon dioxide emissions while promoting the economic prosperity of EU countries. Indeed, many works have been done in this field, remarking on climate change’s impacts on economies and the need for determinant environmental policies inside the EU. This paper investigates the effect of climate change on economic growth using nonlinear dynamic panel methods for 15 countries of the EU in the period 1981–2019. Specifically, it is examined the impact of temperature, precipitation, and CO_2_ emissions on economic growth. So, autoregressive distributed lags (ARDL) methods were employed, overcoming cross-dependency and also considering linearity and nonlinearity. The results showed that economic growth has positive nonlinear relationship with long-run temperature, but in short-run they have a symmetric negative association. Moreover, precipitation has long-run negative and a short-run positive relationship with economic growth. However, when CO_2_ emissions are added, then model’s performance is decreased, and precipitation has a positive effect on economic growth, but all others, except from temperature increase, become insignificant. Finally, actions should be taken for more stable climate conditions and consistent environmental policies by EU countries.

**Graphical abstract:**

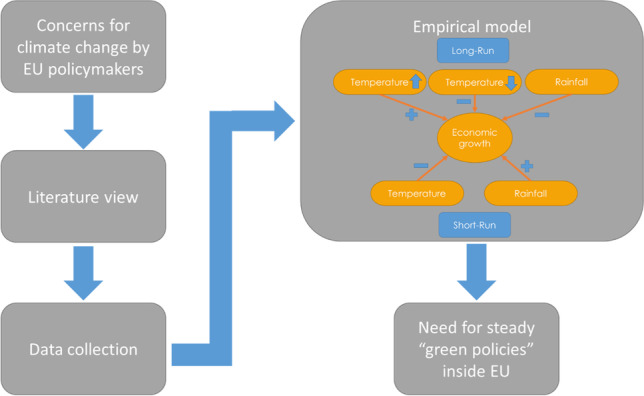

## Introduction

It is an indisputable fact that production and economies are affected by climate conditions, from the very first years of human existence. Especially, in Europe’s economic history, the utilization of land and agricultural had played crucial role in human development and surviving. For example, harsh fluctuations on temperature or water availability could lead to reduced harvest and thus increased mortality rates (Erdkamp et al. 2021). Although, even in modern economies of EU, production of vital agricultural goods is expected to be negatively affected by climate change (Knox et al. 2016). This is an alarming fact because it could possibly drive into market equilibrium collapse, raising up socioeconomic crises (EEA 2021).

Nowadays, the reduction of carbon dioxide pollution as a result of climate change is a crucial objective in the European Union, accompanied by a plethora of environmental policies (Cifuentes-Faura 2022). For instance, “European Climate Law” (2021/1119) is targeted in limiting levels of net greenhouse gas emissions by at least 55% in 2030, compared to ones of 1990, and finally achieving net zero levels by 2050, in a cost-effective way (European Climate Law 2021). Synergy in this attempt between European Union and United Nations in Tokyo Protocol (1998) and Paris Agreement (2015) is also remarkable. The governance quality of each EU country is still found as a key factor for the efficiency of environmental policy applications (Apergis and Garćıa 2019).

Despite the environmental awareness of EU policies, it is widely accepted that European continent is more and more affected by climate change. For example, Pfeifer et al. (2019) found that heat shocks, summer precipitation extremes, higher summer temperatures, and greater differences between day and night temperatures are dealt in highly populated European regions. Additionally, there are concerns about increased greenhouse emissions in the future by the high economic European activity, unless drastic ecological measures are taken in supply chain (Giannakis and Zittis 2021).

Taking into account these, an effort is made in this study for a dynamic contribution in resolving this problematic situation by the investigating the effect of climate change on economic growth at EU level. Both symmetric and asymmetric dynamic panel methods are employed, considering for cross-dependency so the impact of temperature, rainfall, and CO_2_ emissions per capita on economic growth be addressed. The results showed that economic growth has positive relationship with long-run temperature in a nonlinear way, but in short-run they have a symmetric negative association. Precipitation has long-run negative and a short-run positive relationship with economic growth. However, when CO_2_ emissions are added, precipitation has a positive effect on economic growth, but all others, except from temperature increase, become insignificant. Finally, actions should be taken for more stable climate conditions and consistent environmental policies by EU countries.

This work contributes to current literature because it is the first attempt to estimate the asymmetric impact of climate change through time on a panel sample of EU economies using high-resolution climate data. Moreover, it validates the risks of climate instability from 1981 to 2019 on the economic level in EU countries. Therefore, the results produced are crucial to be taken into account by both EU policymakers and EU member states’ central governments, requiring even more eco-friendly actions to be taken immediately.

In the “Literature” section, some fundamental previous works about the effect of climate change on economic growth are presented. Then, in the “Data” section, the strategy of the empirical models is shown and, in the “Framework and models” section, we have the outcome. Finally, in the “Conclusion” section, the basic results and methods of the work are discussed.

## Literature view

One of the most fundamental recent studies on this field was done by Dell et al. (2008). Specifically, was used annual data for 50 years in worldwide panel data in order to examine the effect of temperature and precipitation on economic growth, using dynamic methods. In conclusion, the deducted temperature increase has little effect on economic growth for financially sound countries, but the negative effect, reduced output level and growth rate, and also economic and political problems for the poor ones.

In more recent literature, Burke et al. (2015) figured out a nonlinear effect of temperature on economic growth using nonlinear fixed-effects (FE) models. Characteristically, productivity was found to be maximized at a temperature of 13 °C and harshly diminished for greater ones. This association has been international since 1960 and is irrelevant to agricultural activities or the development of the countries. Future predictions that were made showed only a slight increase in temperature and more unequal income distribution, and they were interpreted as over-optimistic.

The effect of climate change in three tourism-based countries of Mediterranean Βasin: Greece, Spain, and Turkey, was explored by Du and Ng (2018), employing Ordinary Least Squares and Quantile regression methods. In particular, it was denoted that the negative footprint of temperature increase in these specific countries is greater in comparison with G-7 and a group of developing countries. As a result, there is now a need for international collaboration for these three countries, so as to overcome the negative reverberations of climate change for their economies.

Heterogeneous dynamic models were applied by Sequeira et al. (2018) and revealed that increasing temperature has not reduced economic growth for about the last 50 years at the international level. It should be noted that special attention was paid to the global correlated effects between countries. Lastly, it was inferred that lower-income countries and countries with hot and temperate climates are in benefit from precipitation increments. Nevertheless, a negative outcome from rises in temperature was shown for the first ones and from the increments of precipitation in the countries with a colder climate.

One other study in the effects of climate on economic growth, focused mainly on precipitation, was done by Kotz et al. (2022). For this reason, 1554 regions globally were analyzed for a period of 40 years. Specifically, the distribution of rainfall at several timeframes and their macroeconomic effects. From econometric aspect, panel regional and annually FE regressions were estimated. Eventually, that daily rainfall extremes produced from human activities have detrimental negative impacts on worldwide economies. Therefore, the need for re-evaluate of the disadvantages of climate change is recognized.

In addition, numerous surveys on this field were concentrated in Africa and Sub-Saharan countries, employing panel ARDL methods. For example, in work of Lanzafame (2014) analyzed the effect of temperature and precipitation on economic growth for Africa continent, using data from 1962 to 2000 for 36 African countries. It was observed low significance of precipitation on economic growth and vulnerability on weather changes. Hence, corrective measures are needed to be taken in these cases.

Later, a nonlinear impact of temperature on economic growth of Sub-Saharan countries was identified by Alagidede et al. (2016). In more detail, the relationship between GDP per capita and temperature could be described by a “Laffer curve” (curve with the shape of inverted Latin letter U), where economic growth is increased for temperatures below 24.9 °C but downturned for ones above that value.

One more recent study by Meyghani et al. (2022) was concerned about climate change’s impact on the economies of the Middle East and Northern Africa (MENA). In particular, fully modified ordinary least squares (FMOLS) and dynamic ordinary least squares (DOLS) models were applied, certificating with statistical significance the deterioration of economic growth by both temperature and rainfall increments, at 0.25 and 0.024% (for FMOLS) and 0.28 and 0.005% (for DOLS), respectively.

In the bargain of climate change discussion, the effect of CO_2_ emissions is also the main determinant factor of economic growth. Although, the way that influence is an object of dispute across the scientific community. As a common part of all the research, the requirement for environmental policies by all economic members and in all sectors is observed.

The impact of carbon emissions on GDP was explored by Aslan et al. (2021), using panel quantile regression for 17 Mediterranean countries. Specifically, it was revealed that low levels of economic growth could be escorted countries by low CO_2_ pollution, accomplishing sustainability. However, this outcome was insignificant for higher levels of growth. Also, was noticed a two-way dependency between CO_2_ emissions and GDP.

Besides, the paper of Hongxing et al. (2021) analyzed the interaction between economic growth, energy consumption, urbanization, trade, and CO_2_ pollution in 81 belt and road initiative (BRI) countries, for the period 1990–2018. Particularly, with the use of pooled mean group (PMG) estimations was found that the contribution significance of all other magnitudes on economic growth is different across geographical regions. Additionally, from causality tests, CO_2_ emissions were found as one of the variables responsible for economic growth. In the end, it was recognized the need for fiscal policies from governments that motivate businesses and dwellers in “green” practices and for the use οf renewable energy resources.

In the paper of Iqbal et al. (2022), the influence of CO_2_ pollution, renewable energy, and a group of economic variables on GDP for BRICS countries from 2000 to 2018 was researched. Μore clearly, PMG, mean group (MG), FMOLS, and DOLS were applied, depicting a positive long-run effect of the two environmental variables on economic growth. Therefore, encourage for renewable energy sources consumption should be given in these countries.

The effect of a group of pollution and economic variables (including carbon emissions) on economic growth was analyzed by Yiew et al. (2021) for G-20 countries from 1995 to 2014. The use of PMG and FMOLS estimations confirmed the positive influence of pollution on economic growth, while CO2 pollution had the greatest impact on it. Eventually, the necessity of environmental policies for pollution diminution was declared as an essential action against climate change.

The work of Balsalobre-Lorente and Leitão (2020) examined the relationship between renewable energy, trade, CO_2_ emissions, and international tourism on economic growth for the panel of EU-28 from 1995 to 2014. In the econometric methods utilized were included FMOLS, DOLS, and FE methods, showing a positive association among CO_2_ pollution and economic growth. Finally, the requirement for more intense policies for achieving sustainability in EU is resulted.

Additionally, the nexus between renewable and non-renewable energy consumption, CO_2_ pollution, and economic growth for 26 countries of the EU for the period 1990–2018 was analyzed by Asiedu et al. (2021). This work revealed a significant positive correlation between CO_2_ emissions and economic growth. A negative impact of these emissions on economic growth was found from the use of FMOLS and DOLS models. Moreover, the results of the Granger causality tests that were implemented had been interpreted as inconsistent.

The effect of clean and non-clean energy use on economic growth and pollution from carbon emissions in PIMC countries was scrutinized by Ali et al. (2022) for the period 1980–2019. There, ARDL models were employed, considering both the existence and lack of cross-dependence across countries. As results, economic growth is advantaged by the utilization of both clean and non-clean energy but damaged by CO_2_ emissions. So, further, inspect of outcomes revealed that GDP growth is possible by utilizing non-clean energy.

## Data

The examined sample consists of 15 European countries for the period 1981–2019. So, this is a panel data sample with *N* = 15 cross-sections and *T* = 39 years (balanced panel data), in total of 585 observations.

Specifically, as an economic variable, per capita GDP was computed by using real GDP per country and corresponding population, downloaded from Penn World Table version 10.0 (Feenstra et al. 2015). Per capita GDP is considered an index of economic productivity for each country. In addition, as emissions data was employed, CO_2_ per capita emissions on a production basis were harvested from Our World in Data sector named CO2 and GHG Emissions (Our world in data: project based on global carbon 2020).

As climatic data were used 2 m temperature above the surface level and total precipitation, taken from the ERA5-Land monthly averaged data from 1981 to present, downloaded from Copernicus climate change service in 0.1 × 0.1 gridded resolution (Muñoz Sabater 2019) (about 9 × 9 km area grid cells). This is a re-analysis data-set in which are contained modeled and real observations, offering accurate past climate data. Note that temperature is converted from Kelvin to Celsius degrees by subtracting by 273.15 and precipitation from the monthly average in m to total monthly precipitation in mm by multiplying by month’s days × 1000.

These climatic variables were averaged at the year level and then weighted by the population distribution of each country, as proposed by Dell et al. (2008). For the gridded population, we used data from the Gridded Population of the World project, versions 1, 3, and 4, provided by the Socioeconomic Data and Applications Center (SEDAC) of NASA (CIESIN - Columbia University  et al. 2005; CIESIN - Columbia University 2017, 2018).

### Data presentation

**Fig. 1:**
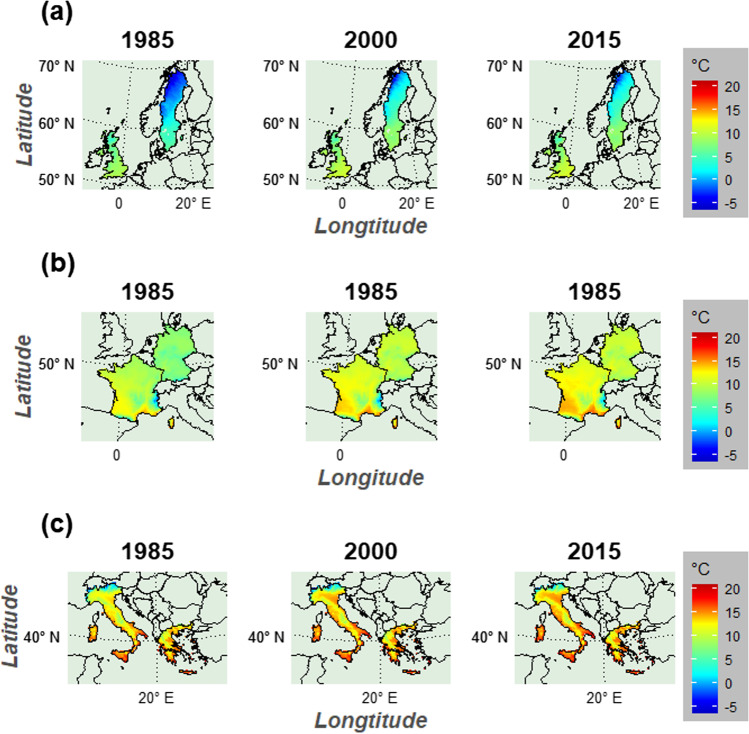
Temperature maps of 1985, 2000, and 2015, for **a** UK and Sweden, **b** France and Germany, and **c** Italy and Greece

Annual average temperature maps for years 1985, 2000, and 2015 for 6 selected countries of this sample, covering most of Europe’s climate zones,^1^ are presented in Fig. 1 (Appendix B). In all of them, the temperature increase is rough, but the differences from 1985 to 2000 are more violent than the ones from 2000 to 2015. Specifically, in Fig. 1a, temperature change is presented for 2 North European countries: the UK—an oceanic climate country and Sweden—a sub-Arctic climate except humid continental climate at the South. Here, the temperature increase is intense for Central and Southern England and London, UK. The same goes for the Scandinavian Mountains and in the South of Sweden. Moreover, The Fig. 1b depicts temperature maps for 2 countries of central Europe: France—with mainly oceanic climate with the Mediterranean at the South shore, and Germany—with an oceanic climate at the west and humid continental climate at the East. In these, it is clear that the only Massif Central and North Pyrenees of France and German Alpes remain unchanged from temperature increase, although the other regions are struck by highly crescent temperatures. Especially, France’s South shore is about 15 °C in 2015. Lastly, Fig. 1c presents 2 South European countries: Italy—mainly Mediterranean climate with a humidly subtropical climate in the North and Greece—mainly Mediterranean climate with a steppe climate in the North-East. In this case, the already hot regions become hotter and Northern Italy and Greece reached even 15 °C in 2015.

**Fig. 2:**
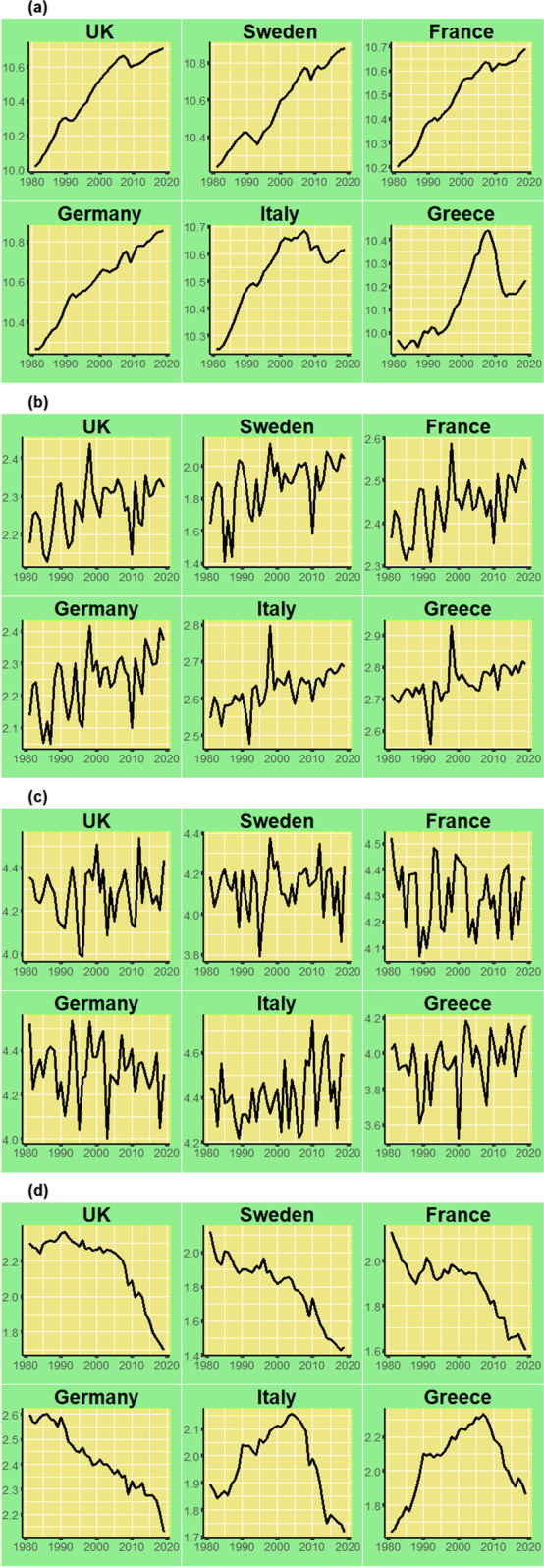
Line graphs of **a** GDP per capita (in log scale), **b** temperature (in log scale and weighted by population), **c** rainfall (in log scale and weighted by population), and **d** CO_2_ emissions per capita (in log scale) for 6 selected countries

Figure 2a (Appendix B) illustrates the real GDP per capita (in log scale), respectively, of these 6 countries for the period for analysis. In all graphs, a positive trend is observed. Additionally, Fig. 2b (Appendix B) displays the temperatures of the same countries (in log scale and weighted by population) for the whole sample period. In this variable, there is consecutive rises and falls, but with a slightly positive trend. Subsequently, Fig. 2c (Appendix B) visualizes the corresponding graphs of precipitation (rainfall). In these, it is shown some symmetry in their progress, but it is also represented as more stable.

The Fig. 2d depicts graphs of per capita CO_2_ production (in log scale). Here, the negative trend is in all countries. However, Italy and Greece (which belong to South Europe) have an upward trend until mid-00, and from then the per capita emission output diminished too. The different process of these two countries was allowed by Kyoto Protocol, as they had low production and emission level (United Nations 1998). But, when high levels of pollution were reached, then it needed to be taken for more strict environmental measures too.

**Fig. 3:**
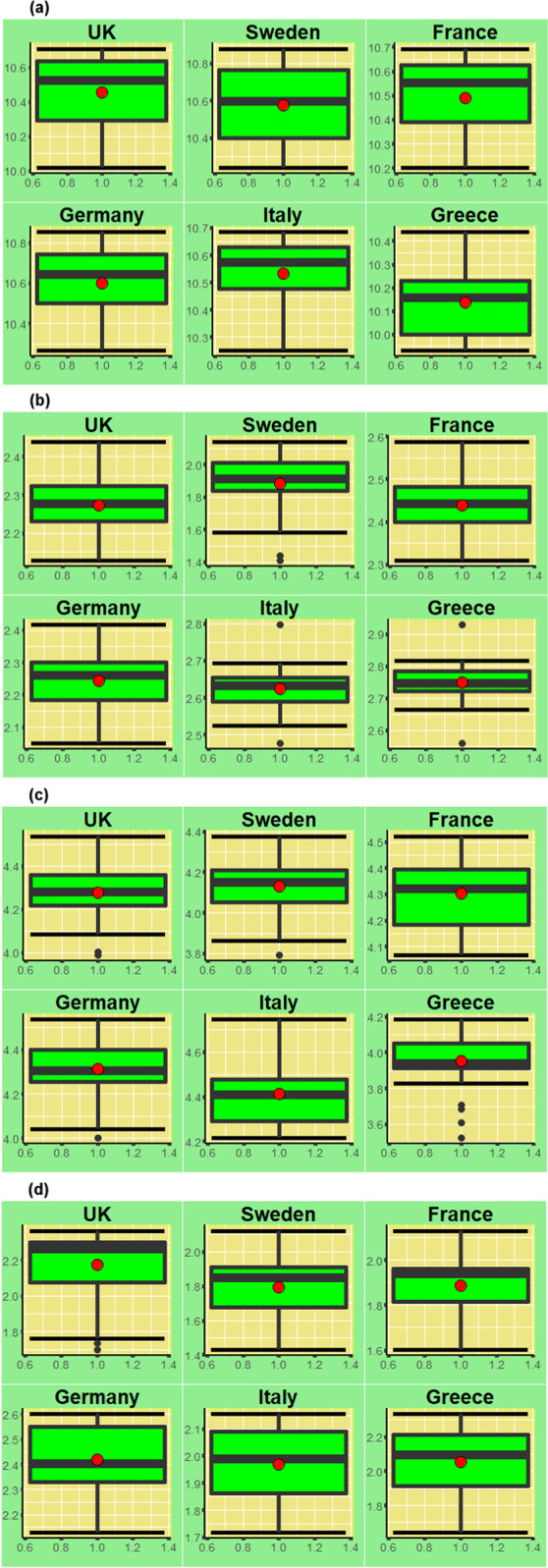
Boxplots of **a** GDP per capita (in log scale), **b** temperature (in log scale and weighted by population), **c** rainfall (in log scale and weighted by population), and **d** CO_2_ emissions per capita (in log scale) for 6 selected countries

Figure 3 (Appendix B) drawn boxplots of the same data. Specifically, in most of them, the mean (red dot) is very close to the median of the boxplot, indicating a possible normal distribution in them and no severe skewness is observed. Moreover, the number of outliers in the majority of the boxplots is very small or non-existing, underlining that most of the observations are associated with the boxplot values. Additionally, boxplots of real GDP per capita and per capita CO_2_ (Fig. 3a and d, respectively) is shown more variability than the ones of temperature and precipitation (Fig. 3b and c).

### Descriptive statistics

Table 1 (Appendix A) presents the basic descriptive statistics of the data that were depicted in the 3.1 (all in logarithmic scale). Here, the normal distribution is followed by the most variables for each country. Exceptions are the temperatures of France, Germany, Italy, and Greece and precipitation of Greece and per capita CO_2_ production of the UK, where the null hypothesis of the normal distribution is rejected.

## Framework and models

### Theoretical framework

This study utilized the Cobb–Douglas relationship that was implied in the paper of Lanzafame (2014), augmented by the effect of CO_2_ emissions per capita on real GDP. Therefore:

1 $$Y_{it}\mathit=A_iTemperature_{it}^aRainfall_{it}^\beta\left(CO_{\mathit2}E\right)_{it}^\gamma L_{it}e^{\varepsilon_{it}}$$

where real GDP is represented by *Y*, temperature by temperature, precipitation by rainfall, CO_2_ emissions per capita by CO_2_E, and the total population of country by *L*, and *A* is technology level of each country (which is assumed as constant). So, for ${{\varvec{y}}}_{{\varvec{i}}{\varvec{t}}}= {{\varvec{Y}}}_{{\varvec{i}}{\varvec{t}}}/{{\varvec{L}}}_{{\varvec{i}}{\varvec{t}}}$, where *y* is real GDP per capita, relationship (1) could be transformed as

2 $${y}_{it}={A}_{i}{\mathrm{Temperature}}_{it}^{{\alpha }_{i}} {\mathrm{Rainfall}}_{it}^{{\beta }_{i}} {\left({\mathrm{CO}}_{2}\mathrm{E}\right)}_{it}^{{\gamma }_{i}}{e}^{{\varepsilon }_{it}}$$

By transforming (2) in log form, the result is

3 $${log(y}_{it})={log(A}_{i})+ {\alpha }_{i}{ log(\mathrm{Temperature}}_{it})+{\beta }_{i}{ log(\mathrm{Rainfall}}_{it}) +{{\gamma }_{i}{ log({\mathrm{CO}}_{2}E}_{it}) +\varepsilon }_{it}$$

And for reasons of abbreviation, (3) will be written as

4 $${\mathrm{GDPPC}}_{it}={k}_{i}+ {\alpha }_{i}{ \mathrm{Temp}}_{it}+{\beta }_{i}{ \mathrm{Rain}}_{it} +{{\gamma }_{i}{ \mathrm{CO}2}_{it} +\varepsilon }_{it}$$

With log(*y*) relabeled as GDPPC, log(A) as *k*, log(temperature) as temp, log(Rainfall) as rain, and CO_2_E as CO2.

### Exploratory of cross-dependence and stationarity

First and foremost, cross-dependence is needed to be tested. So, because of the large dimensions of the sample, the cross-dependence test of Pesaran (2004) is utilized. Then, for testing the stationarity of the data, both cross-independent and cross-dependent unit roots are deployed. First, Levin et al. (2002) (or LLC), Im et al. (2003) (or IPS), and Fisher-type panel ADF and PP unit root tests (Maddala and Wu 2002; Choi 2001) are performed. No cross-dependency is assumed between the countries of each variable in these tests. Note that the common unit root process is supposed in the LLC test and an individual one is considered in the other three. Moreover, as optimal lag length for the test was set by Schwarz information criteria. Secondly, cross-section IPS (CIPS) of Pesaran’s (2007) unit root is used, so as to test for stationarity under the presence of dependency between countries. Here, ADF lag selection was decided via the Akaike information criterion (AIC).

### Panel cointegration tests

In order to acquire a comprehensive view of the examined data, Pedroni (1999) and Kao (1999) panel cointegration tests are conducted. Both of these two tests are based on Engle–Granger (1987) cointegration tests. Here, it should be underlined that Panel ARDL Models are equipped with their specialized cointegration tests.

### Model specification

Due to the large dimensions of the panel data (the number of periods is larger than the number of cross-sections) for the results of stationarity tests in the following section, the pooled mean group/ARDL methods are preferred, according to Asteriou et al. (2020), who used these methods with data with similar behavior. Moreover, other ARDL methods, like mean group and dynamic fixed effects, are based on large *N* asymptotics, and so inconsistent estimations could be produced by their use in a medium *N* sample like this. So, pooled mean group method is supposed to be more consistent than the others, capturing also short-run heterogeneity and long-run homogeneity of the variables slopes and synthesizing both pooling and averaging (Pesaran et al. 1999; Favara 2003).

#### Panel ARDL model

The panel autoregressive distributed lags models are used in the absence of cointegration of other tests, I(1) dependent variable and mixed stationarity—I(0) and I(1)—independent variables, as proposed by Pesaran et al. (1999). This dynamic model is based on the idea that the value of dependent variable in each specific is affected by its previous values, the independent variables and their lags.

In the case of this study, it could be depicted by transforming (4) as

5 $$\begin{array}{c}{\mathrm{GDPPC}}_{it}=k_i+\sum\limits_{j=1}^pa_{ij}{\mathrm{GDPPC}}_{i,t-j}+\sum\limits_{j=0}^qb_{ij}{\mathrm{Temp}}_{i,t-j}+\\\sum\limits_{j=0}^qc_{ij}{\mathrm{Rain}}_{i,t-j}+\sum\limits_{j=0}^qd_{ij}{\mathrm{CO}2}_{i,t-j}+\varepsilon_{it}\end{array}$$

Also, *i* = 1,.., 15 indicates each of the 15 countries, *p* is the number of GDPPC lags used, and *q* is the number of lags of independent variables. It should be noted that the optimum number of lags is selected using AIC.

For estimating the short-run coefficients, the error correction form of this model is conducted. In this case, the short-run model is the (5) estimated in first differences and augmented by the residuals of the (5):

6 $$\begin{array}{l}\Delta {\mathrm{GDPPC}}_{it}={k}_{i}+{\varphi }_{i}\left({\mathrm{GDPPC}}_{i,t-j}-{\theta }_{1}{\mathrm{Temp}}_{i,t-j}-{\theta }_{2}{\mathrm{Rain}}_{i,t-j}-{\theta }_{2}{\mathrm{CO}2}_{i,t-j}\right)\\ +\sum\limits_{j}^{p-1}{\lambda }_{ij}{\mathrm{\Delta GDPPC}}_{i,t-j}+\sum\limits_{j}^{q}{{\lambda }^{'}}_{ij}{\mathrm{\Delta Temp}}_{i,t-j}\\ +\sum\limits_{j}^{q}{{\lambda }^{''}}_{ij}{\mathrm{\Delta Rain}}_{i,t-j}+\sum\limits_{j}^{q}{{\lambda }^{'''}}_{ij}{\mathrm{\Delta CO}2}_{i,t-j} +{\varepsilon }_{it}\end{array}$$

Or, by expanding the parenthesis:

7 $$\begin{array}{l}{\Delta \mathrm{GDPPC}}_{it}={k}_{i}+{\varphi }_{i}{\mathrm{GDPPC}}_{i,t-j}-{\gamma }_{i}{\mathrm{Temp}}_{i,t-j}-{{\gamma }^{'}}_{i}{\mathrm{Rain}}_{i, t-j}-{{\gamma }^{''}}_{i}{\mathrm{CO}2}_{i, t-j}\\ +\sum \limits_{j}^{p-1}{\lambda }_{ij}{\mathrm{\Delta GDPPC}}_{i,t-j}+\sum\limits_{j}^{q}{{\lambda }^{'}}_{ij}{\Delta \mathrm{Temp}}_{i,t-j}+\sum\limits_{j}^{q}{{\lambda }^{''}}_{ij}{\mathrm{\Delta Rain}}_{i, t-j}\\ +\sum\limits_{j}^{q}{{\lambda }^{'''}}_{ij}{\mathrm{\Delta CO}2}_{i, t-j}+{\varepsilon }_{it}\end{array}$$

where ${\theta }_{1}$ and ${\theta }_{2}$ are estimations of (7) of the independent variables and ${\gamma }_{i}={\varphi }_{i}*{\theta }_{1}$ and ${{\gamma }^{^{\prime}}}_{i}={\varphi }_{i}*{\theta }_{2}$. Note that ${\varphi }_{i}$ represents the speed of adjustment of short-run variables to the long-run relationship. Hence, a negative and statistically significant ${\varphi }_{i}$ signals cointegration of the variables. But, in case this coefficient is statistically insignificant, then there is no long-run cointegration in the model. Moreover, the long-run coefficients should be tested via the Wald test whether their coexistence is statistically significant and so they indeed have a simultaneous impact on the dependent variable.

#### Panel ARDL model augmented with common correlated effects

In the ARDL model in (5), it is assumed cross-section independence of the variables. As a result, in the presence of cross-dependency, computing inconsistent and biased estimators. Hence, one solution to this problem was presented by Pesaran (2006) by using a common correlated effects procedure. According to this methodology, there are some unobserved factors that could be approximated by augmenting (5) with the cross-sectional averages of the dependent and independent variables. Furthermore, the estimators produced from this model are correct even in the existence of autocorrelation in its residuals (Pesaran 2006). Unfortunately, no economic interpretation of these averages could be extracted.

Therefore, adding cross-sectional averages in (5) for each used variable there is:

8 $$\begin{array}{c}{\mathrm{GDPPC}}_{it}={k}_{i}+\sum\limits_{j=1}^{p}{a}_{ij}{\mathrm{GDPPC}}_{i,t-j}+\sum\limits_{j=0}^{q}{b}_{ij}{\mathrm{Temp}}_{i,t-j}+\sum\limits_{j=0}^{q}{c}_{ij}{\mathrm{Rain}}_{i,t-j}+\sum\limits_{j=0}^{q}{d}_{ij}{\mathrm{CO}2}_{i,t-j}\\ {+ \beta }_{i}\sum\limits_{j=0}^{p}{\overline{\mathrm{GDPPC}} }_{t}+{{\beta }^{'}}_{i}\sum\limits_{j=0}^{q}{\overline{\mathrm{Temp}} }_{t}+{{\beta }^{''}}_{i}\sum\limits_{j=0}^{q}{\overline{\mathrm{Rain}} }_{t}+{{\beta }^{'''}}_{i}\sum\limits_{j=0}^{q}{\overline{\mathrm{CO}2} }_{t}+{\varepsilon }_{it}\end{array}$$

#### Panel nonlinear ARDL model

This model is based on the hypothesis that the result of the dependent variable would be different from the increase and from the decrease of one independent variable, as it was proposed by Shin et al. (2014). In order to avoid multicollinearity problems, the increase and the decrease of the target variable and here temperature are expressed as

9 $${\mathrm{Temp}}_{i, t}^{+}=\sum\limits_{j=1}^{t}\Delta {\mathrm{Temp}}_{t}^{+}=\sum\limits_{j=1}^{t}\mathrm{max}({\mathrm{\Delta Temp}}_{t},0)$$

10 $${\mathrm{Temp}}_{i, t}^{-}=\sum\limits_{j=1}^{t}\Delta {\mathrm{Temp}}_{t}^{-}=\sum\limits_{j=1}^{t}min({\mathrm{\Delta Temp}}_{t},0)$$

Therefore, the model (5) is converted into

11 $${\mathrm{GDPPC}}_{it}={k}_{i}+\sum\limits_{j=1}^{p}{a}_{ij}{\mathrm{GDPPC}}_{i,t-j}+\sum\limits_{j=0}^{q}{b}_{ij}{\mathrm{Temp}}_{i,t-j}^{+}+\sum\limits_{j=0}^{q}{{b}^{^{\prime}}}_{ij}{\mathrm{Temp}}_{i,t-j}^{-}+\sum\limits_{j=0}^{p}{c}_{ij}{\mathrm{Precip}}_{i,t-j}+\sum\limits_{j=0}^{p}{d}_{ij}{\mathrm{CO}2}_{i,t-j}+{\varepsilon }_{it}$$

It should be underlined that the Wald test must be performed in this case, both in the short and in the long horizon, in order to confirm the existence of asymmetry.

## Results

### Basic tests

The first issue is cross-dependence between countries for each variable. In Table 2 (Appendix A), strong evidence of statically cross-dependence for all variables is presented. This is a highly expected result because of the geospatial and time-aggregated nature of the data.

The outputs of both cross-independent and cross-dependent unit root tests are presented in Tables 3 and 4 (Appendix A), respectively. By the highest majority of them, it is agreed that GDP per capita and CO_2_ emissions are stable in first differences, I(1), but temperature and rainfall are stationary at levels, I(0).

Continuing the analysis, no cointegration is shown by Pedroni (1999) and Kao (1999) cointegration tests in Table 5. Hopefully, it will not be a probleme, as long as special cointegration tests are available for panel ARDL methods.

### Estimated models

In all cases, there were estimated using two different groups of independent variables. In the first one, per capita GDP is affected only by temperature and rainfall. But, in the second one, the CO_2_ effect is included too. Additionally, as the first step in all estimations, the trend and intercept are included. However, if the trend was statistically insignificant, it was removed from the model, increasing its AIC. Thereafter, in case that intercept was statistically insignificant, it was abstracted from the model, increasing its AIC too. Additionally, the Wooldridge autocorrelation test for the residuals is included in every model, as it was proposed by Wooldridge (2010). Moreover, lag selection is performed by using AIC.

#### ARDL models

In Table 6 (Appendix A), the results of the basic ARDL model without and with including CO_2_ variable are presented. In the long run and in both cases, GDP per capita is significantly and positively affected by an increase in temperature, but without CO_2_ addition, the rainfall is insignificant and with it is acquired a significant positive effect. Additionally, the existence of long-run impact for these two models is verified by the Wald test. It should be noted that standard errors of the coefficients become lower when including CO_2_ emissions, indicating more consistent coefficients when pollution is added. Moreover, both speeds of adjustment are significant, but their magnitude is higher when including CO_2_ (22.3% versus 3.7%).

In the short-run, economic growth is positively influenced by its past values in both models. In addition, real GDP per capita is negatively affected by temperature when not adding CO_2_ emissions, but this impact is not significant with the presence of CO_2_ pollution. Reversely, influence of rainfall is insignificant, but when emissions included gets significant with positive impact on economic growth. Moreover, pollution is seemed to have positive effect on economic growth.

Besides, residuals are characterized by stationarity, and have no Wooldridge autocorrelation, pointing out no spurious results in both models. But, cross-dependence is statistically significant, showing that the results are awkward. Despite these issues, higher AIC has resulted when including CO_2_.

#### ARDL-CCE models

In Table 7 (Appendix A), the results of ARDL models when including common correlated effects without and with adding CO_2_ variable are summarized. Specifically, in the long-run, rain has a significant increment effect on per capita GDP, but temperature is insignificant when abstracting CO_2_ and has a significant negative effect on growth when adding the emissions’ impact. Additionally, CO_2_ seems to have a positive impact on economic growth. Note that the long-run impact of each of these groups of variables is significant. In addition, in both models, cointegration is statistically significant, but its speed is higher in the one without CO_2_ (14% against 7.5%).

In the short-run, economic growth seems to be promoted by its preview values, but when adding pollution’s impact, a negative effect is appeared too. Also, a negative impact of temperature on real GDP per capita is observed when CO_2_ emissions are omitted. Nevertheless, all other short-run influences in both models are statistically not significant.

At the same time, the errors produced from both models are corrected in their cross-correlation (as expected), are not time-correlated, and they are stable. Besides these, higher AIC is returned putting in emissions’ effect rather than omitting it.

#### NARDL models

The Table 8 (Appendix A) illustrates results of nonlinear ARDL models. In the long-run, temperature decrease is statistically significant and has a negative impact on economic growth of the countries. But the temperature increment has a positive effect on GDP per capita only in the absence of CO_2_; otherwise, this effect becomes insignificant. Additionally, a positive effect on economy is expected to come up when emissions levels are increased. Furthermore, speed of adjustment is significant for both cases, although that it is faster when including emissions’ effects (7.9% versus 21.8%). Moreover, the standard errors of coefficients are greater when excluding impact of emissions, showing that a more consistent model is resulted by its existence.

However, for both cases, in the short run, the hypothesis of symmetry cannot be rejected. Besides, temperature decrease is significant, with a positive impact on economic growth. Although, when including emissions temperature’s rise and fall and rainfall become insignificant, but emissions’ variable is significant with a positive effect on economic growth. In addition, the dependent variable is positively associated with its previous values in the two models. However, when adding CO_2_ emissions, a positive trend has resulted, but a negative one has occurred when it is omitted.

It should be highlighted that residuals of both models are outlined by cross-sectional dependency, stationarity, and no autocorrelation. Although, AIC is greater when CO_2_ pollution is included.

### NARDL-CCE models

Table 9 (Appendix A) presents the outputs for nonlinear ARDL models, applying common correlated effects. In this specific occasion, the results when including CO_2_ emissions and excluding them are much different.

On one hand, without the CO_2_ variable and in the long run, a temperature increase shock results in higher GDP per capita, but a decrease has a higher (in absolute values) negative impact on economic growth. It is needed to be highlighted that the existence of this asymmetry is supported by the corresponding Wald test. Furthermore, a long-run increase in rainfall has a negative effect on economic growth. Moreover, a significant speed of adjustment is indicated by the cointegration term and is equal to 5.6% Also, the past lags of economic growth have a positive impact on GDP per capita. In the short-run and without adding the CO_2_ emission effect, both the rise and fall of the temperature are statistically significant and show a negative impact on economic growth, but symmetric (according to the Wald test), and growth is increased by rainfall positive shocks.

On the other hand, if CO_2_ emissions are included, they have positive association with economic growth, which is significant in the long-run but insignificant in the short run. Besides, a significant and positive effect of rainfall on dependent variable is observed both in the short and the long run. Temperature increase has positive and significant effect on economic growth, but its fall is insignificant. Adjustment term is statistically insignificant, so no cointegration is accomplished and the null of asymmetry for short and long run is rejected.

Lastly, it should be underlined that errors produced from both models are neither cross nor time correlated and they are stable. Despite these, AIC is higher on model without CO_2_.

## Conclusion

**Fig. 4:**
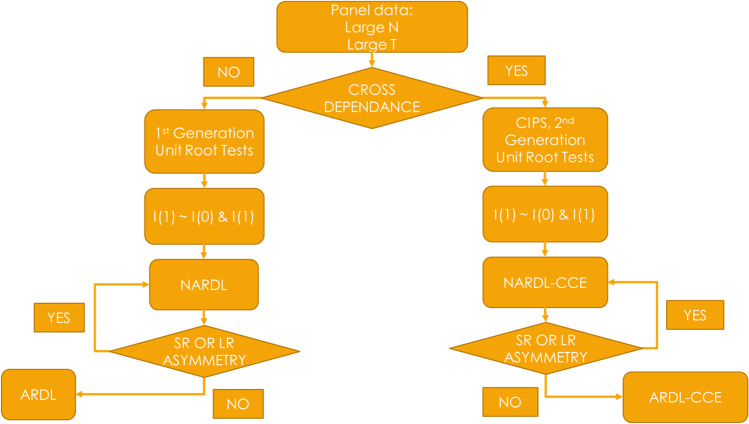
Econometric methodology framework

**Fig. 5:**
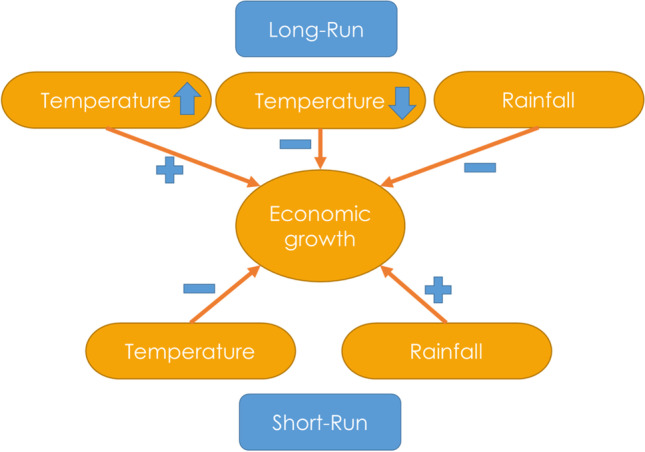
NARDL-CCE synopsis illustration

The Table 10 (Appendix A) depicts the summary of models presented, with their AIC sorted in increasing order. According to this, the nonlinear ARDL model, which counts for CCE, seems to have the best adjustment than all other models. The econometric methodology that was followed is depicted in Fig. 4 (Appendix B), and results of best-fitted NARDL-CCE model are illustrated in Fig. 5 (Appendix B).

This work is deduced that economic growth is increased by temperature rise but decreased by a temperature fall with more impact in growth (in absolute terms). Therefore, past works denoting the existence of a nonlinear relationship between economic growth and temperature (Burke et al. 2015; Alagidede et al. 2016) are certified. Hence, the complexity of impact of temperature changes on GDP growth is more highlighted because it is much more than a simple linear relationship.

So, short-term temperature decreases are expected to help economies’ situation the year they take place. Indeed, sudden heatwaves in Europe are found to decrease these countries’ GDP by about 0.3–0.5% (García-León et al. 2021). The fact that the short-run temperature effect is harmful but the long-run one is positive could be explained by the ability of countries to adapt to disastrous situations in the short run to the long run (Alagidede et al. 2016).

Despite these, economic growth is promoted by short-run rainfall shocks. This could be because of the water sufficiency that is occurring, dodging drought danger in the country. But, in the long run, GDP per capita is decreased by long-run rainfall increases, which is reasonable, as long as this magnitude is weighted by population, and therefore, extreme rainfall could be responsible for damages in towns that ought to be repaired in the long-run. This long-run negative association is also depicted in some of the most recent literature views (Kotz et al. 2022; Meyghani et al. 2022).

Causes of this climate variable’s behavior could be explained by the review of Abbass et al. (2022). According to this, economies are threatened by long-term impacts of climate crisis on agricultural production and so nutrition of populations and their total yielding, on disrupting structures of traditional ecosystems and animals’ survival, on multiplying pathogenic infections and on tourism sector by distorting destinations’ image.

Every time that CO_2_ emissions were included, a positive relationship with economic growth occurred in harmony with the many of the previous literature (Balsalobre-Lorente and Leitão 2020; Yiew et al. 2021; Hongxing et al. 2021; Iqbal et al. 2022). However, a concern is that temperature and rainfall decrease are insignificant when pollution is joined, model has lower AIC, and there is no cointegration.

In the selected NARDL-CCE model, 3 lags for the climate variables as regressors were chosen by AIC (Table 9, Appendix A). From this, it could be extracted that every time economic growth is directly influenced by climate information of the past 3 years. Consequently, every time an environmental policy is applied, it should be consistent for at least 3 years, so the results of this policy can be observable. Although, due to the autoregressive nature of the model, it would certainly take even more years of consequent “green policies” to create sustainable economic growth in the EU.

This existence of lagged impact of climate change on economies has many extensions. An example is a work of Mukherjee and Ouattara (2021), where a worldwide panel sample inflation is statistically significantly affected by temperature shocks even for 4 years after they occur. Besides, the study of Atsalakis et al. (2021) proved that economies are influenced by natural disasters up to 5 years after they happen, raising also concerns about the multiply of them due to the increasing impacts of galloping climate change.

As a result, environment cleaning is a slow and long-term procedure with dangerous economic impacts if not taken seriously. So, the EU’s persistence in combining both economic prosperity and intense attempts for environmental improvement and cleaning (Cifuentes-Faura 2022) is fully justified. Hopefully, the COVID-19 pandemic taught that even a short period of a massive reduction of fossil energy consumption is capable of great environmental benefits, including aerial health, aquatic health, and ecology (Naseer et al. 2022).

Based on the aforementioned, it is an indisputable fact that actions of acquiring a more environmentally “friendly” policy should always be one of the prime goals of the European Union, despite the great steps that have been made since its establishment. That is not only because a more stable climate is needed inside EU borders (Fagerberg et al. 2016) but also because climate change caused by developed countries encloses a “moral hazard,” damaging the undeveloped ones (Tietenberg and Lewis 2012). In conclusion, a more stable climate is preferred because the position of poor countries gets even worse when global temperature is increased (Dell et al. 2008; Lanzafame 2014; Sequeira et al. 2018) and the short-run situation of the EU itself. As a result, collaboration across EU countries should be even stronger and more organized, without excluding any country.

Consequently, selected policies should be in line with the conclusions of the most recent and notable scientific literature, so as to eco-efficiency and economic growth go together. For example, it is established by Chen et al. (2022) that cleaner production, decreased energy intensity, and sustainable practice of renewable energy resources are likely to create a circle of cost reduction and competitive advantages. Additionally, policies should be more appealing for renewable energy investment, replacement of fossil energy resources with renewable ones (Rafei et al. 2022), development of environmentally friendly technology (Jahanger et al. 2022; Rafei et al. 2022) and include relevant structural changes (Balsalobre-Lorente et al. 2022).

Moreover, measures for carbon emissions reductions should not stop there. For instance, natural resources and environmental pollution could be decreased by proper improvement of human assets, GDP per capita levels, and in some cases, globalization and financial development (Jahanger et al. 2022). In addition, there are specimens where carbon pollution is achieved by the reduction in heating energy consumption intensity, in heating demand, and replacing carbon heating energy sources with less carbon ones (Jiang et al. 2022).

Nevertheless, this study faces some limitations. A key one is that climatic data used are not real observations, but the outcome of reanalysis data without any missing values. So, aspects of their reliability are controversial among meteorologists, as long as some believe that they diverge from real data (Velikou et al. 2022), but others trust them as attested and sufficient approximations (Ben Hamouda et al. 2021). So, future scientists who want to use high-resolution climatic data should consider the trade-off between using real data with missing values against utilizing fully available reanalysis products.

Many interesting extensions of this work could be done. Specifically, the outcome of the model utilized could vary for different continents, the number of countries, countries’ syntheses (e.g., high-income vs low-income countries), and at the worldwide level. Furthermore, another idea is to include additional climatic variables with direct economic impact, like sea waves that affect cargo ships’ travel and wind that powers wind turbines. Finally, employing other econometric models could be quite insightful too.

## Data Availability

Data, coding, command line text, and graphs are available upon request.

## Appendix A

**Table 1:** Descriptive statistics for 6 selected countries

Real GDP per capita (log scale)																							
		UK			Sweden				France				Germany			Italy						Greece	
Mean		10.453			10.573				10.489				10.600			10.533						10.135	
Median		10.525			10.597				10.552				10.643			10.573						10.157	
Maximum		10.707			10.878				10.693				10.852			10.684						10.438	
Minimum		10.020			10.240				10.203				10.267			10.251						9.930	
Std. dev		0.213			0.203				0.153				0.178			0.130						0.155	
Skewness		− 0.551			− 0.034				− 0.498				− 0.412			− 0.937						0.422	
Kurtosis		1.983			1.552				1.873				2.080			2.711						2.030	
Jarque–Bera		3.653			3.413				3.671				2.479			5.848*						2.685	
Temperature (log scale)																							
			UK			Sweden				France				Germany			Italy				Greece		
Mean			2.272			1.882				2.440				2.243			2.624				2.750		
Median			2.277			1.915				2.442				2.261			2.633				2.747		
Maximum			2.439			2.141				2.588				2.416			2.796				2.928		
Minimum			2.129			1.412				2.310				2.050			2.477				2.559		
Std. dev			0.069			0.171				0.066				0.094			0.055				0.057		
Skewness			− 0.257			− 1.083				− 0.108				− 0.360			0.151				− 0.202		
Kurtosis			2.690			3.726				2.554				2.449			4.894				6.403		
Jarque–Bera			2.272			1.882				2.440*				2.243*			2.624*				2.750*		
Precipitation (log scale)																							
				UK			Sweden				France				Germany				Italy	Greece			
Mean				4.275			4.130				4.302				4.312				4.412	3.953			
Median				4.281			4.150				4.321				4.304				4.410	3.940			
Maximum				4.538			4.376				4.521				4.535				4.746	4.183			
Minimum				3.989			3.794				4.068				4.001				4.215	3.523			
Std. dev				0.123			0.120				0.124				0.129				0.134	0.150			
Skewness				− 0.324			− 0.654				− 0.217				− 0.434				0.520	− 0.900			
Kurtosis				2.977			3.626				1.852				3.063				2.574	3.790			
Jarque–Bera				0.684			3.420				2.449				1.231				2.054	6.278*			
Per capita CO_2_ (log scale)																							
	UK							Sweden				France			Germany			Italy					Greece
Mean	2.173							1.792				1.888			2.420			1.968					2.054
Median	2.262							1.847				1.939			2.403			1.989					2.094
Maximum	2.363							2.120				2.131			2.600			2.154					2.333
Minimum	1.701							1.432				1.604			2.129			1.717					1.640
Std. dev	0.190							0.183				0.132			0.125			0.134					0.199
Skewness	− 1.293							− 0.643				− 0.701			− 0.143			− 0.290					− 0.490
Kurtosis	3.330							2.359				2.632			2.195			1.803					2.177
Jarque–Bera	11.042***							3.355				3.412			1.186			2.878					2.659

Significant in *** = 1%, ** = 5%, * = 10% level.

**Table 2:** CD tests

Variable	CD-TEST
GDPPC	60.764***
Temp	46.274***
Rain	14.440***
CO2	31.312***

**Table 3:** Unit root tests (cross-independency assumed)

	Constant	Constant and trend	Constant	Constant and trend	Constant	Constant and trend	Constant	Constant and trend
Test/variable	LLC		IPS		FISHER-ADF		FISHER-PP	
GDPPC	− 6.322***	− 0.798	− 1.483	2.041	38.939	14.977	45.874**	6.386
Temp	− 14.468***	− 17.315***	− 10.458***	− 13.056***	160.889***	194.443***	157.828***	206.909***
Rain	− 22.014***	− 20.197***	− 20.521***	− 19.025***	356.594***	306.379***	412.381***	685.827***
CO2	2.307	2.485	3.573	5.130	14.331	7.033	16.729	7.991
Δ(GDPPC)	− 9.545***	− 8.728***	− 9.930***	− 8.841***	154.191***	128.846***	153.544***	178.485***
ΔTemp	-	-	-	-	-	-	-	-
ΔRain	-	-	-	-	-	-	-	-
ΔCO2	− 19.029***	− 19.568***	− 19.082***	− 20.231***	319.037***	372.347***	333.368***	879.612***

**Table 4:** Unit root tests (cross-dependency assumed)

	Constant	Constant and trend
Test/variable	CIPS	
GDPPC	− 2.129	− 2.113
Temp	− 4.187***	− 4.309***
Rain	− 5.187***	− 4.803***
CO2	− 1.898	− 2.253
ΔGDPPC	− 2.977***	− 3.193***
ΔTemp	-	-
ΔRain	-	-
ΔCO2	− 4.008***	− 3.422***

**Table 5:** Cointegration tests

	Intercept		Trend and intercept	
	*T*-statistic	(Weighted)	*T*-statistic	(Weighted)
Panel v-statistic	− 3.555	− 3.315	11.436***	11.553***
Panel rho-statistic	3.306	2.038	2.487	2.318
Panel PP-statistic	3.474	1.327	1.654	1.214
Panel ADF-statistic	3.358	1.210	2.122	1.536
Group rho-statistic	3.696		3.194	
Group PP-statistic	3.176		1.723	
Group ADF-statistic	3.012		1.493	
Kao	− 0.832			

**Table 6:** ARDL models with and without including impact of CO^*2*^ (standard errors in parentheses)

		ARDL	ARDL (CO2)
Long–run	Temp	1.547*** (0.271)	0.131*** (0.049)
	Rain	0.098 (0.166)	0.099*** (0.028)
	CO2	-	0.377*** (0.020)
Short–run	Cointegration term	− 0.037*** (0.006)	− 0.223*** (0.021)
	Δ(GDPPC (− 1))	0.348*** (0.048)	0.372*** (0.043)
	Δ(Temp.)	− 0.023* (0.012)	0.001 (0.012)
	Δ(Rain)	− 0.002 (0.003)	− 0.010** (0.005)
	Δ(CO2)	-	0.132*** (0.041)
	Constant	0.237*** (0.036)	1.933*** (0.183)
	Trend	-	0.004*** (0.000)
	Number of observations	555	555
	AIC	− 4.681	− 4.883
	Wald test	16.872***	122.653***
	CD-test	31.673***	24.364***
	WD-AR (1)	1.072	0.673
	Residuals	I(0)	I(0)

**Table 7:** ARDL-CCE models with and without including impact of CO^*2*^ (standard errors in parentheses)

		ARDL–CCE	ARDL (CO2)–CCE
Long run	Temp	0.000 (0.050)	− 0.370*** (0.040)
	Rain	0.194*** (0.038)	0.196*** (0.039)
	CO2	-	0.061* (0.034)
Short run	Cointegration term	− 0.140** (0.060)	− 0.075*** (0.026)
	Δ(GDPPC (− 1))	0.647*** (0.077)	0.682*** (0.098)
	Δ(GDPPC (− 2))	− 0.133 (0.086)	− 0.206** (0.080)
	Δ(GDPPC (− 3))	0.162** (0.080)	0.032 (0.061)
	Δ(Temp.)	0.022** (0.038)	0.004 (0.033)
	Δ(Temp (− 1))	0.016 (0.039)	0.035 (0.031)
	Δ(Temp (− 2))	0.015 (0.032)	0.034 (0.026)
	Δ(Temp (− 3))	− 0.035** (0.014)	-
	Δ(Rain)	− 0.002 (0.018)	− 0.002 (0.013)
	Δ(Rain (− 1))	− 0.005 (0.008)	0.008 (0.010)
	Δ(Rain (− 2))	− 0.017 (0.014)	− 0.007 (0.006)
	Δ(Rain (− 3))	− 0.019 (0.013)	-
	Δ(CO2)	-	− 0.011 (0.029)
	Δ(CO2 (− 1))	-	0.064 (0.042)
	Δ(CO2 (− 2))	-	0.001 (0.039)
	Constant	− 0.061** (0.027)	−
	Number of observations	525	525
	AIC	− 5.524	-5.478
	Wald test	13.220***	1038.517***
	CD-test	0.445***	− 0.332***
	WD-AR (1)	1.184	0.312
	Residuals	I(0)	I(0)

**Table 8:** NARDL models with and without including impact of CO2 (standard errors in parentheses)

		NARDL	NARDL (CO2)
Long run	Temp^+^	1.730*** (0.315)	0.197 (0.058)
	Temp^−^	0.614* (0.317)	0.131** (0.065)
	Rain	0.009 (0.082)	0.083*** (0.028)
	CO2	-	0.366*** (0.023)
Short run	Cointegration term	− 0.079*** (0.016)	− 0.218*** (0.023)
	Δ(GDPPC (− 1))	0.343*** (0.048)	0.376*** (0.043)
	Δ(Temp^+^)	− 0.038 (0.024)	0.016 (0.020)
	Δ(Temp^−^)	− 0.059** (0.025)	− 0.017 (0.028)
	Δ(Rain)	0.003 (0.004)	− 0.006 (0.005)
	Δ(CO2)	-	0.129*** (0.042)
	Constant	0.800*** (0.157)	1.975*** (0.207)
	Trend	− 0.001*** (0.000)	0.003*** (0.000)
	Number of observations	555	555
	AIC	− 4.820	− 5.005
	Wald test short-run	0.316	0.636
	Wald test long-run	18.147***	1.333
	CD-test	29.980***	23.720***
	WD-AR (1)	0.770	0.428
	Residuals	I(0)	I(0)

**Table 9:** NARDL-CCE models with and without including impact of CO^*2*^ (standard errors in parentheses)

		NARDL–CCE	NARDL (CO2)–CCE
Long run	Temp^+^	1.435*** (0.159)	0.215* (0.126)
	Temp^−^	1.818*** (0.195)	− 0.098 (0.147)
	Rain	− 0.448*** (0.064)	0.088* (0.047)
	CO2	-	0.604*** (0.049)
Short run	Cointegration Term	− 0.056** (0.028)	− 0.030 (0.020)
	Δ(GDPPC (− 1))	0.435*** (0.091)	0.382*** (0.063)
	Δ(GDPPC (− 2))	− 0.042 (0.075)	− 0.091** (0.042)
	Δ(GDPPC (− 3))	0.030 (0.040)	0.027 (0.040)
	Δ(Temp^+^)	− 0.115* (0.063)	− 0.006 (0.022)
	Δ(Temp^+^(− 1))	− 0.075 (0.056)	0.010 (0.032)
	Δ(Temp^+^ (− 2))	0.067 (0.069)	-
	Δ(Temp^−^)	− 0.160* (0.093)	0.010 (0.035)
	Δ(Temp^−^ (− 1))	− 0.026 (0.064)	− 0.036 (0.031)
	Δ(Temp^−^ (− 2))	− 0.023 (0.041)	-
	Δ(Rain)	0.031*** (0.011)	0.015* (0.008)
	Δ(Rain (− 1))	0.018 (0.009)	0.012* (0.007)
	Δ(Rain (− 2))	0.002 (0.008)	-
	Δ(CO2)	-	0.036 (0.032)
	Δ(CO2 (− 1))	-	0.045 (0.033)
	AIC	− 5.991	− 5.603
	Trend	0.001*	
	Number of observations	525	525
	Wald test short-run	0.040	0.836
	Wald test long-run	10.222***	84.721***
	CD-test	− 0.359	0.744
	WD-AR (1)	− 0.600	1.019
	Residuals	I(0)	I(0)

**Table 10:** AIC model selection

Rank	Model	AIC
1st	NARDL-CCE	− 5.991
2nd	NARDL(CO2)-CCE	− 5.603
3rd	ARDL-CCE	− 5.524
4th	ARDL(CO2)-CCE	− 5.478
5th	NARDL(CO2)	− 5.005
6th	ARDL(CO2)	− 4.883
7th	NARDL	− 4.820
8th	ARDL	− 4.681

## Footnotes

1 For more about recent Köppen climate classifications, see Beck et al (2018).

## Materials availability

Data, coding, command line text, and graphs are available upon request.
